# Quantitative Analysis of Objective Forward Scattering and Its Relevant Factors in Eyes with Cataract

**DOI:** 10.1038/s41598-019-39680-7

**Published:** 2019-02-28

**Authors:** Kazutaka Kamiya, Fujimura Fusako, Kawamorita Takushi, Masahide Takahashi, Wakako Ando, Yoshihiko Iida, Nobuyuki Shoji

**Affiliations:** 1 Visual Physiology, School of Allied Health Sciences, Kitasato University, Tokyo, Japan; 2 Department of Ophthalmology, Faculty of Medicine, Kitasato University, Tokyo, Japan

## Abstract

This study was aimed to quantitatively assess objective forward scattering and its relevant factors in eyes having cataract. Our study comprised 192 eyes of 192 patients (mean age, 71.3 ± 9.2 (standard deviation) years) who have cataract formation for surgical consultation. We determined uncorrected and corrected distance visual acuities (UDVA and CDVA), manifest refraction, the grade of nuclear sclerosis, objective scattering index (OSI) with the OQAS II (Visiometrics, Spain), log(s) with the C-Quant (Oculus, Germany), and ocular higher-order aberrations (HOAs) using the wavefront sensor (KR-1W, Topcon, Japan). The mean OSI was 5.11 ± 3.19 (0.90 to 20.90). We found explanatory variables relevant to the OSI to be, logMAR CDVA (p < 0.0001, partial regression coefficient B = 5.917) and log(s) (p = 0.0006, B = 0.911) (adjusted R^2^ = 0.333), in order of influence. No significant correlation was found with other clinical factors such as gender, age, manifest refraction, UDVA, ocular HOAs, or nuclear sclerosis. Eyes with worse CDVA and higher log(s) are more predisposed to show higher OSI in cataractous eyes. It is suggested that objective forward scattering was associated, not only with CDVA, but also with subjective forward scattering, in cataractous subjects.

## Introduction

Cataract has widely been recognized as one of the common eye diseases which degrade retinal image quality, and thus deteriorate visual performance in daily practice. Both light scatter and aberration play a vital role in visual quality in cataractous eyes. Considering that forward light scattering directly affects this quality compared with backward light scattering, it is especially important to quantify this forward scattering in a clinical setting.

Currently there are two commercially available devices for the evaluation of forward scattering in clinical use. The point spread function meter (Optical Quality Analysis System II (OQAS), Visiometrics, Terassa, Spain) makes an objective assessment based on the double-pass method^1,2^. The straylight meter (C-Quant, Oculus, Wetzlar, Germany) makes a subjective assessment based on the compensation method^3,4^. We previously showed that the OQAS measurement provides a better reproducibility than the C-quant measurement, possibly due to the independence of subjective responses and the shorter time required for the measurements^5^.

Until now, there have been several studies to evaluate this objective forward scattering and its relationship with visual acuity in eyes with cataract^6–12^. However, to the best of our knowledge, the relevant factors for the objective forward scattering in cataractous eyes have so far not been investigated. Moreover, the relationship between subjective and objective forward scattering has not been fully elucidated in a large cohort of cataractous patients. It may give us intrinsic insights on the role of objective light scattering in visual degradation in such patients. The aim of the current study is twofold; to quantitatively evaluate objective forward scattering in eyes having cataract, and to determine the relevant factors influencing this scattering, using single and multiple regression analyses in such eyes.

## Results

Table 1 shows the study demographics. The objective scattering index (OSI) was 5.11 ± 3.19 (range, 0.9 to 20.9). The OSI was 4.62 ± 2.37 (range, 1.3 to 11.2), 5.17 ± 3.19 (range, 0.9 to 16.8), and 5.37 ± 3.73 (range, 1.1 to 20.9), in the cortical, nuclear sclerosis, and posterior subcapsular cataract subgroups. We found no significant differences in the OSI among the three groups (analysis of variance, p = 0.551).

**Table 1:** Demographic data of the study population.

Demographic data	
Number of subjects	192
Age (years)	71.3 ± 9.2 years (range, 26 to 89 years)
Gender (male = 0, female = 1)	M: F = 94: 98
LogMAR UDVA	0.68 ± 0.48 (range, −0.08 to 2)
LogMAR CDVA	0.17 ± 0.21 (range, −0.08 to 1.30)
Manifest refraction (D)	−1.77 ± 3.90 D (range, −19.00 to 3.00 D)
Nuclear Grade	2.0 ± 0.5 D (range, 1 to 4)
OSI	5.11 ± 3.19 (range, 0.90 to 20.90)
log(s)	1.87 ± 1.01 (range, 0.70 to 6.00)
Ocular HOAs (µm, 4 mm)	0.33 ± 0.29 (range, 0.11 to 2.08)
Ocular HOAs (µm, 6 mm)	1.07 ± 1.12 (range, 0.38 to 7.08)

D = diopter, logMAR = logarithm of the minimal angle of resolution, log(s) = log (straylight), OSI = objective scattering index.

**Figure 1:**
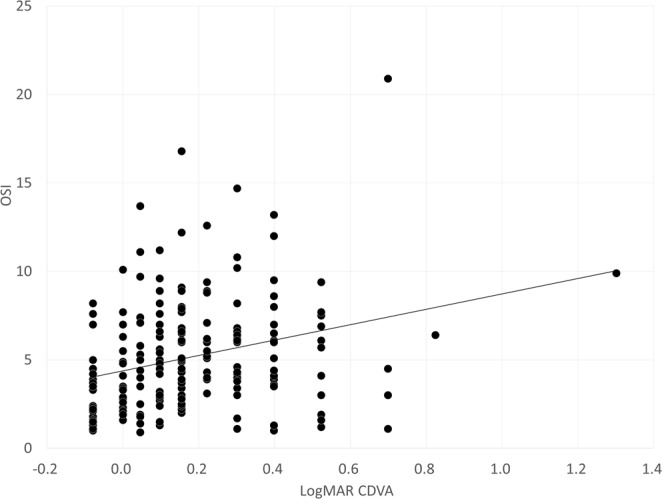
A graph showing a significant correlation between the objective scattering index (OSI) and logarithm of the minimal angle of resolution (logMAR) corrected distance visual acuity (CDVA) (r = 0.328, p < 0.001).

**Figure 2:**
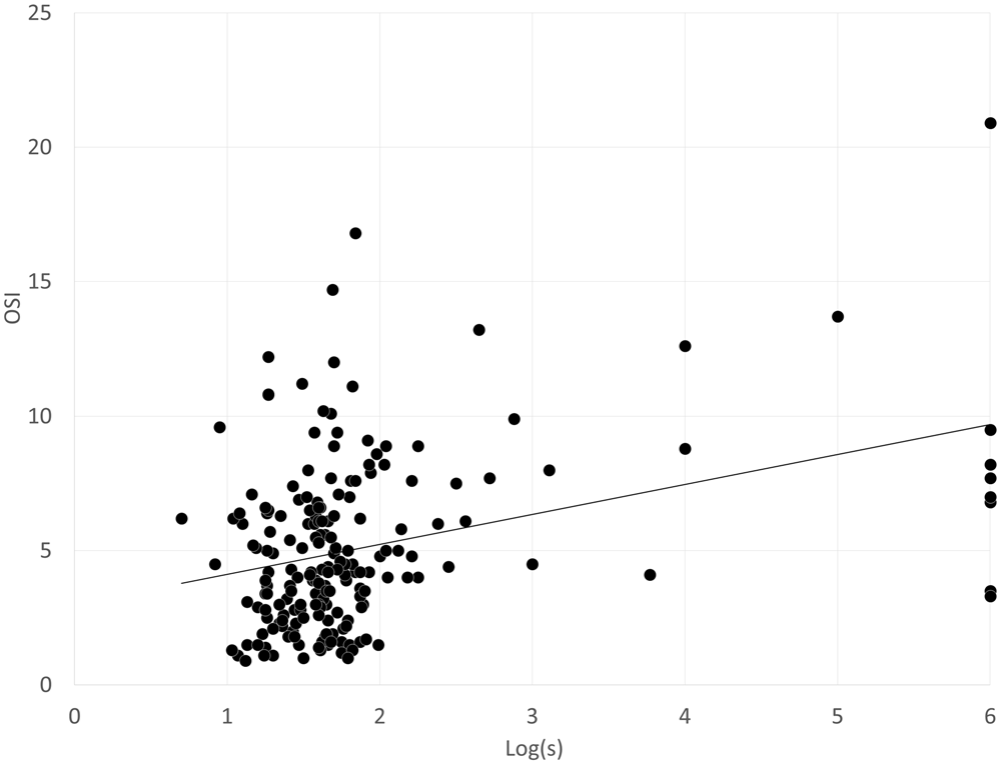
A graph showing a significant correlation between the objective scattering index (OSI) and log(s) (r = 0.309, p < 0.001).

Table 2 shows the multiple regression analysis results. In order of influence, the explanatory variables relevant to the OSI were, logarithm of the minimal angle of resolution corrected distance visual acuity (logMAR CDVA) (p < 0.0001, partial regression coefficient B = 5.917) and logarithmic straylight value (log(s)) (p = 0.0006, B = 0.911) (adjusted R^2^ = 0.333). The multiple regression equation in this study was calculated as follows: OSI = (5.917 × logMAR CDVA) + (0.911 × log(s)) −1.659. No significant association with other clinical factors were found such as gender, age, manifest refraction, uncorrected and corrected distance visual acuity (UDVA), axial length, ocular higher-order aberrations (HOAs), or nuclear sclerosis. In order to determine the magnitude of each variable’s influence, we calculated the standardized partial regression coefficient. The most relevant variable was logMAR CDVA, and log(s) was found to be the second. We found comparable results by using a single rank correlation test as listed in Table 2. The relationships of the OSI with logMAR CDVA and with log(s) can be found in Figs 1 and 2, respectively. The OSI of the study population was significantly higher, with worse CDVA, higher log(s), or both.

**Table 2:** Results of multiple regression analysis to select variables relevant to the objective scattering index (OSI) in eyes with cataract.

Variables	Partial regression coefficient	Standardized partial regression coefficient	P-value
Age (years)	0.036	0.094	0.223
Gender (male = 0, female = 1)	0.713	0.113	0.110
LogMAR UDVA	−0.046	−0.007	0.954
LogMAR CDVA	5.917	0.356	<0.001
Manifest refraction	0.072	0.084	0.470
Nuclear Grade	0.463	0.059	0.400
Log (s)	0.911	0.260	<0.001
Ocular HOAs (µm, 4 mm)	0.541	0.050	0.752
Ocular HOAs (µm, 6 mm)	0.277	0.099	0.532
Constant	−1.659		
Adjusted R² = 0.333			

logMAR = logarithm of the minimal angle of resolution, UDVA = uncorrected distance visual acuity, CDVA = corrected distance visual acuity, log(s) = log (straylight), HOAs = higher-order aberrations.

## Discussion

In this study, our results show that the objective forward scattering was significantly associated with logMAR CDVA, as well as with subjective forward scattering, in eyes having cataract. However, the majority of the variance has not been fully explained, evidence for this is the small R^2^ value. To the best of our knowledge, this is the first study to quantitatively investigate the relevant factors affecting the objective forward scattering in cataractous subjects by applying multiple regression analysis.

So far, there have been several studies on the objective forward scattering and its association with CDVA in eyes having cataract, as listed in Table 3^2,6–12^. Artal *et al*.^2^ firstly introduced the OSI parameter, which was defined as the ratio between the integrated light in the periphery and in the surroundings of the central peak of the double-pass retinal images to classify cataract. Vilaseca *et al*.^6^ found a decrease in CDVA and an increase in the OSI with increasing cataract grade, and a significant nonlinear correlation between OSI and CDVA, although there was a high variability in the results. Cabot *et al*.^7^ showed a mean OSI of 4.1 ± 3.5, and a significant association between the OSI and CDVA in eyes having cataract. Pan *et al*.^8^ demonstrated a significant correlation between OSI and CDVA, and that an OSI of 3.0 or more can be a possible objective cut-off for preoperative decision making for cataract surgery. Galliot *et al*.^9^ and Cochener *et al*.^10^ described that the mean OSI was 4.97 ± 3.13, and that it was significantly associated with CDVA in a large cohort of cataractous patients. Paz Filgueira *et al*.^11^ stated that the OSI was significantly associated not only with CDVA (r = −0.636, p < 0.001), but also with log(s) (r = 0.629, p < 0.001) in eyes with early cataract. Martínez-Roda *et al*.^12^ found significant correlations with LOCS III classification in terms of log(s) and OSI, although they were slightly stronger with OSI for all cataract types. These previous findings were in accordance with our current findings, in terms of a significant association between the OSI and CDVA.

**Table 3:** Previous studies on the objective scattering index (OSI) and its relationship of visual acuity in eyes having cataract.

Author	Year	Eyes	Age	OSI	Correlation with visual acuity	P-value
Artal *et al*.^2^	2011	53	73 ± 5	1 ≤ OSI < 3 (Early), 3 ≤ OSI < 7 (Mature), 7 ≤ OSI (Severe)	N.A.	N.A.
				3.0 ± 1.0 (NO2), 6.0 ± 2.0 (NO3),		
				9.0 ± 3.0 (NO4)		
Vilaseca *et al*.^6^	2012	188	61.7 ± 10.5	2.24 ± 1.34 (NO1), 3.82 ± 1.85 (NO2), 5.74 ± 2.45 (NO3)	r = 0.878 (NO)	N.A.
				2.08 ± 1.02 (C1), 3.64 ± 1.43 (C2), 6.94 ± 0.46(C3)	r = 0.843 (C)	
				1.91 ± 1.10 (P1), 2.96 ± 1.21 (P2), 5.62 ± 1.53 (P3)	r = 0.844 (P).	
Cabot *et al*.^7^	2013	253	68.1 ± 11.1	4.1 ± 3.5	r = 0.4 (≥20/32)	0.001
					r = 0.5 (<20/40)	
Pan *et al*.^8^	2015	60	65.8 ± 7.8	1.77 ± 0.69 (<3.0), 6.30 ± 2.51 (≥3.0)	r = 0.779	<0.001
Galliot *et al*.^9^	2016	1768	72.5 ± 18.2	4.97 ± 3.13	r = −0.47	<0.001
Cochener *et al*.^10^						
Paz Filgueira *et al*.^11^	2016	34	70.0 ± 2.3 (NO1)	1.49 ± 0.51 (NO1), 2.82 ± 1.21 (NO2)	r = 0.629	<0.001
			68.3 ± 4.7 (NO2)	0.89 ± 0.60 (P1), 1.09 ± 0.83 (P2)		
			56.7 ± 5.5 (P1)			
			55.3 ± 4.6 (P2)			
Martínez-Roda *et al*.^12^	2016	78	47 to 86	4.19 ± 3.12 (NO)	r = −0.404 (NO)	N.A.
				4.28 ± 2.12 (C)	r = −0.375 (C)	
				5.20 ± 3.99 (P)	r = −0.477 (P)	
Current		192	71.3 ± 9.2	5.11 ± 3.19	r = 0.328	<0.001

The grading was according to the LOCS III and based on nuclear opalescence (NO1, NO2, NO3, NO4), cortical cataracts (C1, C2,C3), and posterior subcapsular cataracts (P1, P2, P3, P4).

There are at least three limitations to this study. First of all, this was a retrospectively conducted study. The authenticity of our results might be confirmed by conducting a randomized, controlled study which may provide further information. Second, we only included cataractous subjects in whom we could measure all optical parameters with these devices. Accordingly, the study population might be biased, since severe cases who were not measurable for these parameters, such as mature cataract, were excluded from this study. Third, we used trial glasses to correct refractive errors, since the uncorrected refractive error directly affects the outcomes of forward scattering. Therefore, we cannot refute the possibility that the trial glasses induced some additional scattering in this study.

In summary, this study may support the idea that eyes with worse CDVA and eyes with higher log(s) show higher OSI in eyes with cataract. According to our experience, it is indicated that the objective forward scattering is significantly associated with visual acuity as well as with the subjective forward scattering in such subjects.

## Methods

### Study Population

The study protocol was registered with the University Hospital Medical Information Network Clinical Trial Registry (000034758). This retrospective study included one hundred ninety-two eyes of the 192 consecutive patients (94 men and 98 women), who completed all optical examinations at Kitasato University Hospital for cataract surgery consultation. Any concomitant eye diseases such as severe dye eye, corneal degeneration, uveitis, glaucoma, vitreous opacification, macular disease, and trauma, except for cataract, were excluded from the study. We only enrolled one eye per subject at random for statistical analysis. This retrospective review of clinical charts followed the tenets of the Declaration of Helsinki and was approved by the Kitasato University Institutional Review Board. The Institutional Review Board waived the requirement for informed consent.

### Assessment of Refraction, Visual Acuity, and Nuclear Sclerosis

Experienced optometrists used a Snellen chart at a distance of 5 m to measure visual acuity, and an automated refractometer (ARK-700A, Nidek, Gamagori, Japan) as a starting point for a full manifest refraction. The grade of nuclear sclerosis of the crystalline lens was assessed by cataract specialists according to the Emery-Little classification. For subgroup analysis, the cataract type was divided into 3 subgroups (nuclear sclerosis, cortical, and posterior subcapsular cataract), based on slit-lamp biomicroscopy after mydriasis. Since it was challenging to exactly classify the type of cataract, we determined as cases those subjects who presented with advanced forms of 1 of the 3 cataract types, regardless of the concomitant presence of the remaining 2 cataract types.

### Assessment of Objective and Subjective Forward Scatterings and Higher-Order Aberrations

We used the OQAS point spread function meter to assess the OSI, as a measure of objective forward scattering. On the basis of the double-pass technique, this instrument directly measures the point spread function of the optical system for a 4-mm pupil^1^. The OSI is calculated as the ratio between the integrated light in the periphery (between 12 and 20 minutes of arc) and a circular area of 1 minute of arc around the central peak of the double-pass image^2^.

We used the C-Quant straylight meter to assess retinal straylight, as a measure of subjective forward scattering^3^. A test field that consists of a dark circle divided into two semicircles and is surrounded by a ring-shaped flickering light. A counter-phase compensation light is presented in one of the semicircles, reducing the flicker perception on that side. The patient is asked to select which side is flickering more intensely. This process is repeated several times with different levels of compensation light, resulting in log(s). The measurement was approved only when the estimated standard deviation was <0.08 and the quality factor was >1.00^4^.

We used the Hartmann-Shack aberrometer (KR-1W, Topcon, Tokyo, Japan) to assess total ocular HOAs, as the root-mean-square of the third- and fourth-order coefficients, for 4-mm and 6-mm pupils.

### Statistical analysis

We conducted stepwise multiple regression analysis to evaluate the association between various variables and the objective forward scattering. OSI was set as the dependent variable. Explanatory variables in this study included patient age and gender, logMAR UDVA and CDVA, manifest refraction, the grade of nuclear sclerosis, log(s), and ocular HOAs (for 4-mm and 6-mm pupils). We also used the Pearson’s rank correlation test to assess the relationships of the OSI with other variables. One-way analysis of variance was used for the analysis of the OSI among the 3 cataract subgroups. Commercially available statistical software was used to perform the statistical analysis (Bellcurve for Excel, Social Survey Research Information Co, Ltd., Tokyo, Japan). Results are indicated as mean ± standard deviation, and a p-value < 0.05 was deemed statistically significant.
